# Three Cases of Tickborne *Francisella tularensis* Infection, Austria, 2022 

**DOI:** 10.3201/eid2911.230460

**Published:** 2023-11

**Authors:** Florian Heger, Stefanie Schindler, Sonja Pleininger, Astrid Fueszl, Marion Blaschitz, Kathrin Lippert, Patrick Hyden, Peter Hufnagl, David Mutschlechner, Thomas Gremmel, Erwin Hofer, Mateusz Markowicz, Alexander Indra

**Affiliations:** Austrian Agency for Health and Food Safety, Vienna, Austria (F. Heger, S. Schindler, S. Pleininger, A. Fueszl, M. Blaschitz, K. Lippert, P. Hyden, P. Hufnagl, E. Hofer, M. Markowicz, A. Indra);; Cardiology and Intensive Care Medicine, Mistelbach, Austria (D. Mutschlechner, T. Gremmel);; Institute of Cardiovascular Pharmacotherapy and Interventional Cardiology, St. Pölten, Austria (T. Gremmel);; Paracelsus Medical University of Salzburg, Salzburg, Austria (A. Indra)

**Keywords:** Francisella tularensis, tularemia, tick bites, tickborne disease, vector-borne infections, parasites, re-emerging disease, bloodstream infection, arthropod, Austria, Europe

## Abstract

Tularemia is increasing in Austria. We report *Francisella tularensis* subspecies *holarctica* isolated from 3 patients who had been bitten by arthropods. Next-generation sequencing showed substantial isolate similarity. Clinicians should consider bloodstream *F. tularensis* infections for patients with signs/symptoms of ulceroglandular tularemia, and surveillance of potential vectors should be intensified.

Tularemia is a zoonotic disease of the Northern Hemisphere, caused by the highly virulent bacterium *Francisella tularensis.* Although *F. tularensis* subspecies *tularensis* (type A, found only in North America) is associated with severe infections, *F. tularensis* subsp. *holarctica* (type B, found throughout the Northern Hemisphere) causes less severe illness (*1*,*2*). Infection occurs after contact with infected animals, transmission via arthropod vectors, or contact with contaminated water or soil (*3*). Only sporadic infections, detected primarily by serologic testing, have been reported in Austria; therefore, genomic data are scarce (*3*,*4*). Recently, cases of tularemia have increased in Austria (Figure 1). We report 3 tularemia cases that developed after arthropod bites in Austria. Ethics approval was not necessary because routine data were processed in the study and personal data were anonymized. The patients gave written consent for publication of the case reports.

**Figure 1:**
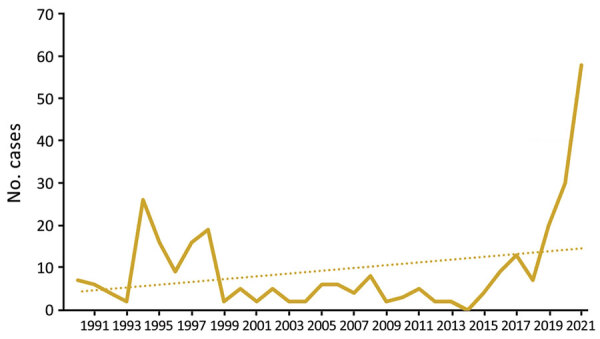
Reported cases (solid line) and trends (dotted line) of tularaemia in Austria, 1990–2021. Data from the Austrian Epidemiologic Reporting System (https://oecd-opsi.org).

## The Cases

Patient 1 was a 64-year-old woman who had received a tick bite in the right inframammary region. After experiencing fever, chills, and a general feeling of discomfort, along with nausea and vomiting, she sought emergency care when symptoms persisted. Clinical examination detected a 20-mm skin lesion surrounded by a reddish hem and central necrotic ulcer. Results of serologic testing for Lyme borreliosis were negative. Stab incision of the skin lesion was performed, and no bacteria grew in culture of the sampled pus. Blood cultures grew *F. tularensis*. Antimicrobial treatment with oral ciprofloxacin (500 mg 2×/d) led to sign/symptom resolution.

Patient 2 was a 5-year-old girl seen at a pediatric outpatient clinic for retroauricular pain after a tick bite 9 days earlier. Clinical examination showed a swollen and reddened pinna, a pus-covered 5-mm retroauricular skin lesion with a central ulcer (Appendix Figure), and swollen cervical lymph nodes. After 2 days, fever, increased lymph node swelling, and tonsillitis developed, and the retroauricular wound deteriorated. Therapy was switched from oral amoxicillin/clavuanic acid (250 mg 4×/d) and fucidin ointment to intravenous ceftriaxone (520 mg 1×/d), followed by oral cefixim (64 mg 2×/d), which alleviated signs and symptoms. A wound swab sample culture yielded *F. tularensis*. Antimicrobial treatment, changed to oral ciprofloxacin (250 mg 2×/d), led to substantial signs/symptom improvement.

Patient 3 was a 76-year-old man who was hospitalized after reporting fever, generalized discomfort, nausea, and vertigo. A red, painless skin lesion with central crust on the left thigh, which probably developed after an arthropod bite, and inguinal lymphadenopathy were noted. Antimicrobial treatment with ampicillin/sulbactam was started because of suspected pneumonia. Eight days after hospitalization, *F. tularensis* grew on anaerobic blood culture. Therapy with ciprofloxacin was initiated and later switched to oral doxycycline (100 mg 2×/d) because of potential allergic reaction to ciprofloxacin, followed by substantial sign/symptom improvement. The findings suggest local transmission of *F. tularensis* after an insect or tick bite.

We used multiplex PCR (Analyticon Instruments GmbH, https://www.analyticon.eu/de) to test samples, and all were positive for *F. tularensis.* Real-time PCR (TIB MolBiol, https://www.tib-molbiol.de), performed for subspecies determination according to manufacturer instructions, yielded *F. tularensis* subsp. *holarctica* (*5*). We determined MICs for 6 antimicrobials by Etest (bioMérieux, https://www.biomerieux.fr) and interpreted results according to Clinical and Laboratory Standards Institute guidelines (*6*) (Table). Because of high-level erythromycin resistance, we assigned the isolates to *F. tularensis* subsp. *holarctica* biovar II (genotype B.12), which was later confirmed by whole-genome sequencing (WGS) to be subclade B.34/B.35.

**Table:** Antimicrobial MICs for *Francisella tularensis* isolates recovered from 3 patients with tickborne tularemia, Austria*

Patient	MIC, mg/L					
	Gentamicin	Erythromycin	Ciprofloxacin	Tetracycline	Doxycycline	Streptomycin
1	0.25	**>256**	0.008	0.047	0.094	1.5
2	0.5	**>256**	0.012	0.032	0.19	3
3	0.19	**>256**	0.008	0.125	0.125	0.75

*Boldface indicates antimicrobial resistance according to Clinical and Laboratory Standards Institute standards (*6*).

**Figure 2:**
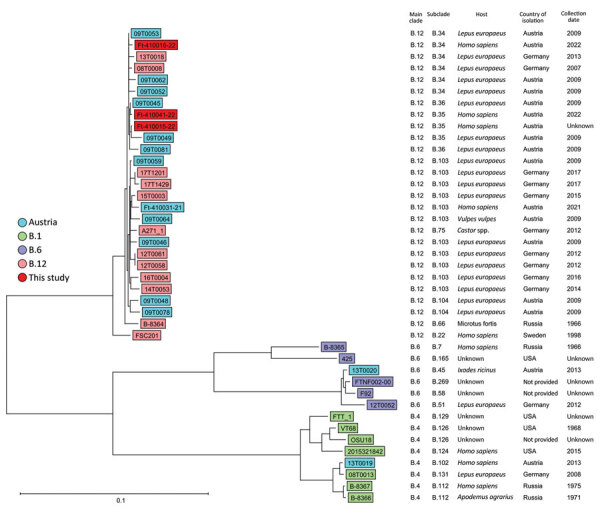
Ridom SeqSphere+ NJ tree (Ridom Gmbh, https://www.ridom.de) for 41 *Francisella tularensis* samples isolated from 3 patients in Austria, based on 1,147 columns from *F. tularensis* core-genome multilocus sequence typing. Scale bar indicates nucleotide substitutions per site. Metadata are provided in the Appendix Table.

For all *F. tularensis* samples, we performed WGS on a NextSeq 2000 instrument (Illumina, Inc., www.illumina.com), 150-bp paired-end, by using a QIAGEN MagAttract HMW DNA Kit (https://www.qiagen.com) for DNA isolation and a Nextera XT DNA Library Prep Kit (Illumina, Inc.) for library preparation. We assembled whole-genome sequences de novo by using SPAdes (*7*) version 3.15.2, analyzed them by using Jspecies webserver (*8*), and corroborated real-time PCR species results by using the TCS (Templeton, Crandall and Sing) calculation method. In a pairwise comparison, the 3 isolates differed in 5 genes: 410015–22 vs. 410016–22 (FTL_0160, FTL_0920, FTL_1212, and FTL_1567); 410015–22 vs. 410041–22 (FTL_0414 and FTL_0920), and 410016–22 vs. 410041–22 (FTL_0160, FTL_0414, FTL_1212, and FTL_1567). Average allelic distance is 3.3 alleles, which is above cluster threshold for this species (1 allele difference). We applied core-genome multilocus sequence typing analysis by using Seqshpere+ version 8.5.1 (Ridom GmbH, https://www.ridom.de) with the published core-genome multilocus sequence typing scheme (*9*) to compare our isolate genomes against genomes from Germany (Bavaria) and Austria from a previous study (PRJEB40963 [*10*]). We identified clades/subclades by using CanSNPer2 (*11*). We submitted the following *F. tularensis* subsp. *holarctica* WGS assemblies to the National Center for Biotechnology Information (BioProject PRJNA900077): biosample SAMN31677967 (410015–22, patient 1), accession no. JAPKFK000000000; biosample SAMN31677968 (410016–22, patient 2), accession no. JAPKFJ000000000; and biosample SAMN32382778 (410041–22, patient 3), accession no. JAPZIK000000000 (Figure 2, Appendix).

## Conclusions

Outbreaks of *F. tularensis* subsp. *holarctica* in Europe are linked mainly to infected rodents and hares; mosquitos and ticks serve as arthropod vectors (*3*). Over the past 30 years (1990–2021), a total of 302 human infections have been reported in Austria, with 2 peaks after outbreaks in hares during 1994–1995 and 1997–1998 (*12**–**14*). So far, national reported cases show an upward trend; in 2021 alone, a total of 58 cases were reported (Figure 1).

Biovar I (genotype B.6) is found mainly in western Europe and biovar II (genotype B.12) in northern and eastern Europe (*11*). Most tularemia cases in Austria are associated with hunting hares and skinning carcasses and are caused by *F. tularensis* subsp. *holarctica* biovar II. Recently, ticks have been identified as vectors, and *F. tularensis* subsp. *holarctica* biovar I was detected in Austria in 2016 (*1*,*15*). Under debate is whether pathogenic potential of biovar I may be higher than that of biovar II (*15*).

Of the 3 patients, 2 reported removing a tick after spending time in rural areas; the third did not observe a tick or insect bite but had local skin alterations that were highly comparable to those seen on other patients with tickborne tularemia. Isolate sequences were closely related to isolates from Austrian and German hares (*10*).

Bloodstream infections with *F. tularensis* after arthropod bites are rarely reported in Europe. One reason might be that in Europe only *F. tularensis* subsp. *holarctica* is found, and it is known to cause milder disease than *F. tularensis* subsp. *tularensis* in the United States. Thus, we find it remarkable that bacteremia developed in 2 patients in Austria. WGS showed a close relationship between isolates from the patients and isolates found mostly in Germany and Austria, as shown in the neighbor-joining tree for 41 *F. tularensis* complete genomes (Figure 2; Appendix Table), which indicates the wild animal population as a host for *F. tularensis* subsp. *holarctica* biovar II (B.12) and ticks as vectors for tularemia in Austria.

Our findings show that ticks represent underestimated vectors for *F. tularensis* transmission in Austria. Aside from other tickborne diseases endemic to Austria, clinicians should consider tularemia as a cause of signs/symptoms that follow tick bites, especially when combined with fever, enlarged and painful lymph nodes, and skin ulcers. Diagnosis can be achieved by molecular testing. Genomic data are essential for understanding dissemination and invasion of certain genotypes that may cause systemic infections. To confidently declare that certain genetic subpopulations are associated with systemic infections, a larger number of isolates and further research are needed. For that reason, Austria established large-scale monitoring of arthropod vectors for the presence of vectorborne pathogens, including *F. tularensis*, to provide public health authorities with knowledge about infection risk for the exposed population.

Appendix Additional information for 3 cases of tickborne *Francisella tularensis* infection, Austria, 2022.
