# Supplementary material for: Three Cases of Tickborne Francisella tularensis Infection, Austria, 2022
Source: Emerg Infect Dis. 2023 Nov;29(11):2349–52. doi: 10.3201/eid2911.230460 (PMC10617340; doi:10.3201/eid2911.230460)
Supplement: Appendix — Additional information for 3 cases of tickborne Francisella tularensis infection, Austria, 2022. [file 23-0460-Techapp-s1.pdf]

Article DOI: https://doi.org/10.3201/eid2911.230460

EID cannot ensure accessibility for supplementary materials supplied by authors. Readers who have difficulty accessing supplementary content should contact the authors for assistance.

Three Cases of Tickborne Tularemia, Austria, 2022

Appendix

Appendix Table. Overview of metadata of 41 *F. tularensis* subsp. *holarctica* complete genomes imported from NCBI to Ridom SeqSphere+.

Strain	Source Type	Host	Isolation Source	Country of Isolation	Collection Date	Main Clade	Sub-Clade	PubMed ID(s)	Nucleotide Accession(s)	Perc. Good Targets	Contig Count (Assembled)	Approximated Genome Size (Mbases)	Avg. Coverage (Assembled)
FSC201	clinical/host-associated	Homo sapiens		Sweden	1998	B.12	B.22		NZ_CP089549.1	99.9	1	1.9	1
2015321842	clinical/host-associated	Homo sapiens	pleural	USA	2015	B.4	B.124		NZ_CP073125.1	95.8	1	1.9	200
08T0013	clinical/host-associated	Lepus europaeus		Germany	2008	B.4	B.131		NZ_CP058301.1	98.7	1	1.9	150
12T0052	clinical/host-associated	Lepus europaeus		Germany	2012	B.6	B.51		NZ_CP058275.1	97.6	1	1.9	150
A271_1	clinical/host-associated	Castor sp.		Germany	2012	B.12	B.75	27356883	NZ_CP048229.1	99.8	1	1.9	100
B-8364	clinical/host-associated	Microtus fortis		Russia	1966	B.12	B.66		NZ_CP044005.1	99.7	1	1.9	196
B-8365	clinical/host-associated	Homo sapiens		Russia	1966	B.6	B.7		NZ_CP044004.1	98.5	1	1.9	205
B-8366	clinical/host-associated	Apodemus agrarius		Russia	1971	B.4	B.112		NZ_CP044003.1	98.8	1	1.9	248
B-8367	clinical/host-associated	Homo sapiens		Russia	1975	B.4	B.112		NZ_CP044002.1	98.8	1	1.9	212
425			Water	USA		B.6	B.165	25931589	NZ_CP010289.1	98.3	1	1.9	
VT68			Muskrat spleen	USA	1968	B.4	B.126	25931589	NZ_CP010288.1	98.6	1	1.9	
FTT_1				USA		B.4	B.129	25931589	NZ_CP009693.1	98.7	1	1.9	767
F92						B.6	B.58	23405342	NC_019537.1	95.1	1	1.9	
OSU18						B.4	B.126	18927608	NC_017463.1	98.1	1	1.9	
FTNF002-00						B.6	B.269	19756146	NC_009749.1	98.7	1	1.9	
08T0008	clinical/host-associated	Lepus europaeus		Germany	2013	B.12	B.34	32991594	GCA_010378045.1	99.4	103	1.8	
09T0045	clinical/host-associated	Lepus europaeus		Austria	2013	B.12	B.36		GCA_010377935.1	99.6	97	1.8	
09T0048	clinical/host-associated	Lepus europaeus		Austria	2009	B.12	B.104		GCA_010377875.1	99.7	98	1.8	

Strain	Source Type	Host	Isolation Source	Country of Isolation	Collection Date	Main Clade	Sub-Clade	PubMed ID(s)	Nucleotide Accession(s)	Perc. Good Targets	Contig Count (Assembled)	Approximated Genome Size (Mbases)	Avg. Coverage (Assembled)
09T0049	clinical/host-associated	Lepus europaeus		Austria	2009	B.12	B.35		GCA_010377865.1	99.5	98	1.8	
09T0046	clinical/host-associated	Lepus europaeus		Austria	2009	B.12	B.103		GCA_010377845.1	99.4	99	1.8	
09T0052	clinical/host-associated	Lepus europaeus		Austria	2009	B.12	B.34		GCA_010377815.1	99.6	98	1.8	
09T0053	clinical/host-associated	Lepus europaeus		Austria	2009	B.12	B.34		GCA_010377805.1	99.6	98	1.8	
09T0062	clinical/host-associated	Lepus europaeus		Austria	2009	B.12	B.34		GCA_010377765.1	99.0	110	1.8	
09T0064	clinical/host-associated	Vulpes vulpes		Austria	2009	B.12	B.103		GCA_010377745.1	99.6	98	1.8	
09T0059	clinical/host-associated	Lepus europaeus		Austria	2009	B.12	B.103		GCA_010377725.1	99.6	99	1.8	
09T0078	clinical/host-associated	Lepus europaeus		Austria	2009	B.12	B.104		GCA_010377675.1	99.6	99	1.8	
09T0081	clinical/host-associated	Lepus europaeus		Austria	2009	B.12	B.36		GCA_010377605.1	99.6	97	1.8	
12T0058	clinical/host-associated	Lepus europaeus		Germany	2009	B.12	B.103	32991594	GCA_010376485.1	99.7	100	1.8	
12T0061	clinical/host-associated	Lepus europaeus		Germany		B.12	B.103	32991594	GCA_010376435.1	99.7	99	1.8	
13T0018	clinical/host-associated	Lepus europaeus		Germany	2022	B.12	B.34	32991594	GCA_010376365.1	98.1	113	1.8	
13T0019	clinical/host-associated	Homo sapiens		Austria	2022	B.4	B.102		GCA_010376335.1	98.4	99	1.8	
13T0020	clinical/host-associated	Ixodes ricinus		Austria	2017	B.6	B.45		GCA_010376315.1	96.3	153	1.8	
14T0053	clinical/host-associated	Lepus europaeus		Germany	2017	B.12	B.103	32991594	GCA_010375985.1	95.9	148	1.8	
15T0003	clinical/host-associated	Lepus europaeus		Germany	2016	B.12	B.103	32991594	GCA_010375455.1	99.6	100	1.8	
16T0004	clinical/host-associated	Lepus europaeus		Germany	2015	B.12	B.103	32991594	GCA_010375285.1	98.5	97	1.8	
17T1201	clinical/host-associated	Lepus europaeus		Germany	2014	B.12	B.103	32991594	GCA_010375095.1	99.7	98	1.8	
17T1429	clinical/host-associated	Lepus europaeus		Germany	2013	B.12	B.103	32991594	GCA_010375055.1	98.2	123	1.8	
Ft-410015-22	clinical/host-associated	Homo sapiens		Austria	2022	B.12	B.35		Submitted	96.0	230	1.8	
Ft-410016-22	clinical/host-associated	Homo sapiens		Austria	2022	B.12	B.34		Submitted	97.6	220	1.8	
Ft-410041-22	clinical/host-associated	Homo sapiens		Austria	2022	B.12	B.35		Submitted	97.6	176	1.8	
Ft-410031-21	clinical/host-associated	Homo sapiens		Austria	2021	B.12	B.103			94.0	228	1.8	

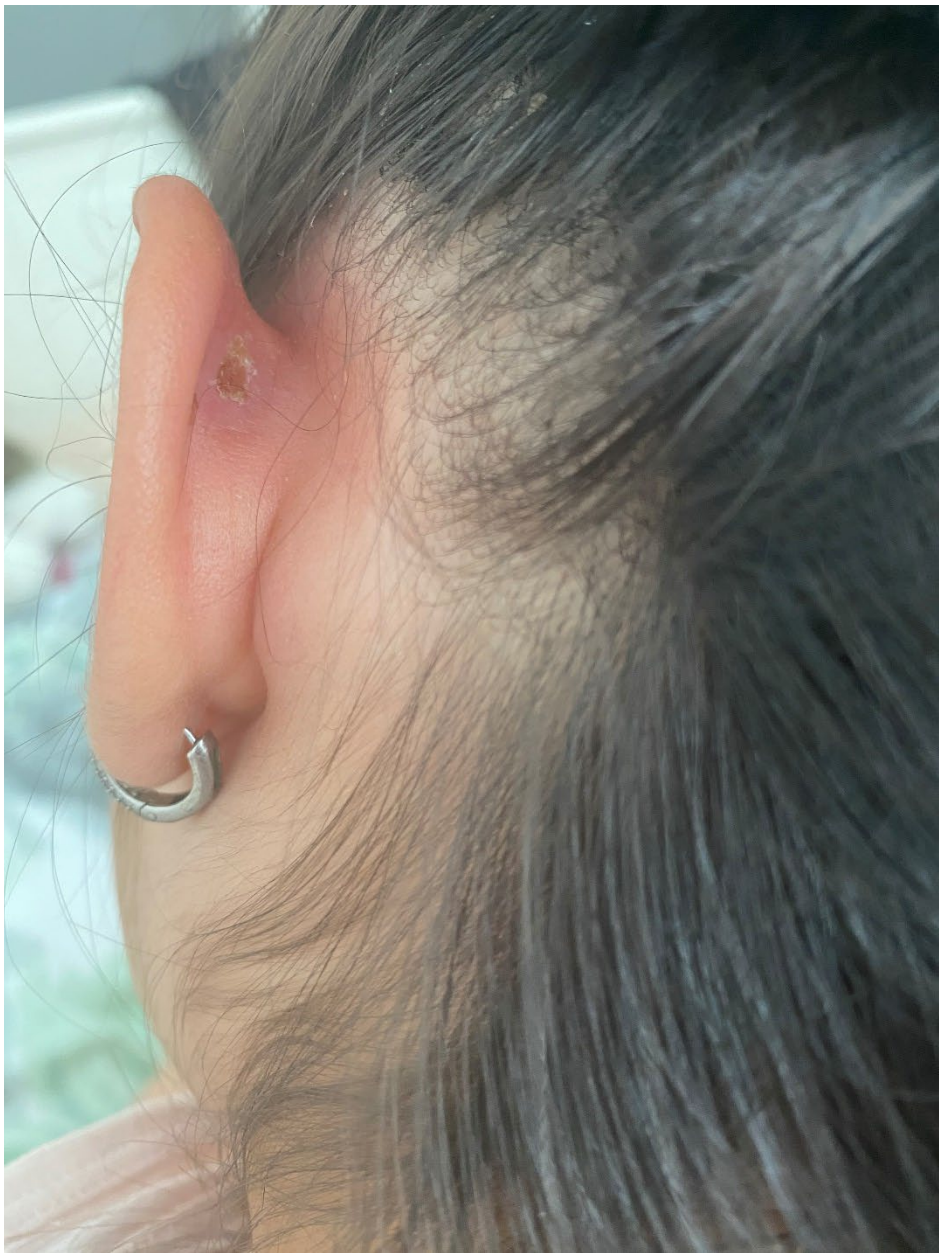

Appendix Figure. Swollen and reddened pinna, with pus-covered 5-mm retroauricular skin lesion with a central ulcer in 5-year-old girl with tick-borne tularemia, Austria.
